# Royal Jelly and *Chlorella vulgaris* Mitigate Gibberellic Acid-Induced Cytogenotoxicity and Hepatotoxicity in Rats via Modulation of the PPARα/AP-1 Signaling Pathway and Suppression of Oxidative Stress and Inflammation

**DOI:** 10.3390/foods12061223

**Published:** 2023-03-13

**Authors:** Sally M. Khadrawy, Doaa Sh. Mohamed, Randa M. Hassan, Mohamed A. Abdelgawad, Mohammed M. Ghoneim, Sultan Alshehri, Nema S. Shaban

**Affiliations:** 1 Zoology Department, Faculty of Science, Beni-Suef University, Beni-Suef 62511, Egypt; 2 Department of Biochemistry and Chemistry of Nutrition, Faculty of Veterinary Medicine, Beni-Suef University, Beni-Suef 62511, Egypt; 3 Cytology and Histology Department, Faculty of Veterinary Medicine, Beni-Suef University, Beni-Suef 62511, Egypt; 4 Department of Pharmaceutical Chemistry, College of Pharmacy, Jouf University, Sakaka 72341, Saudi Arabia; 5 Department of Pharmacy Practice, College of Pharmacy, AlMaarefa University, Ad Diriyah 13713, Saudi Arabia; 6 Pharmacognosy and Medicinal Plants Department, Faculty of Pharmacy, Al-Azhar University, Cairo 11884, Egypt; 7 Department of Pharmaceutics, College of Pharmacy, King Saud University, Riyadh 11451, Saudi Arabia; 8 Department of Pharmacology, Faculty of Veterinary Medicine, Beni-Suef University, Beni-Suef 62511, Egypt

**Keywords:** gibberellic acid, royal jelly, *Chlorella vulgaris*, hepatotoxicity, genotoxicity, PPARα/Ap-1 signaling pathway

## Abstract

Gibberellic acid (GA3) is a well-known plant growth regulator used in several countries, but its widespread use has negative effects on both animal and human health. The current study assesses the protective effect of royal jelly (RJ) and *Chlorella vulgaris* (CV) on the genotoxicity and hepatic injury induced by GA3 in rats. Daily oral administration of 55 mg/kg GA3 to rats for 6 constitutive weeks induced biochemical and histopathological changes in the liver via oxidative stress and inflammation. Co-administration of 300 mg/kg RJ or 500 mg/kg CV with GA3 considerably ameliorated the serum levels of AST (aspartate aminotransferase), ALT (alanine aminotransferase), ALP (alkaline phosphatase), γGT (gamma-glutamyl transferase), total bilirubin, and albumin. Lowered malondialdehyde, tumor necrosis factor α (TNF-α), and nuclear factor κB (NF-κB) levels along with elevated SOD (superoxide dismutase), CAT (catalase), and GPx (glutathione peroxidase) enzyme activities indicated the antioxidant and anti-inflammatory properties of both RJ and CV. Also, they improved the histological structure and reduced cyclooxygenase-2 (COX-2) and inducible nitric oxide synthase (iNOS) expressions along with up-regulating peroxisome proliferator activated receptor α (PPARα) and down-regulating activator protein 1 (AP-1) gene expression. Additionally, chromosomal abnormalities and mitotic index were nearly normalized after treatment with RJ and CV. In conclusion, RJ and CV can protect against GA3-induced genotoxicity and liver toxicity by diminishing oxidative stress and inflammation, and modulating the PPARα/AP-1 signaling pathway.

## 1. Introduction

Plant growth promotors, called phytohormones, are used worldwide in agriculture [1]. The gibberellin hormones represent an important type of phytohormone. Of these hormones, gibberellic acid (GA3) is heavily utilized in Egypt and other countries to increase the growth of many fruits and vegetables by stimulating cell division, flowering, and fruit development to improve the quality of crops [2]. GA3 is largely persistent and stays active in the ground for long periods [3]. Exposure to its residue through consumption of GA3-treated fruits and plants, inhalation of its powder as well as skin contact leads to deleterious effects on human and animal health [4].

GA3 treatment decreases the ability of the cell to scavenge reactive oxygen species (ROS) causing oxidative stress and cell death [5]. ROS attack biomolecules such as DNA, lipids, proteins, and glutathione causing enzyme inactivation, genotoxicity, cell death, and loss of cell membrane functional integrity [6]. GA3 was reported to have genotoxic and cytotoxic effects [7]. Treating cultures of human lymphocytes with gibberellin A3 increased chromosomal abnormalities, sister chromatid exchanges, and DNA mutations [8]. According to Abou-Eisha [9], gibberellic acid triggered a dose-dependent increment in DNA damage in human blood cells. Additionally, GA3 is toxic to many soft organs including the liver, causing alterations in liver enzymes, a disruption in the oxidant/antioxidant balance, and apparent changes in the liver’s architecture [10].

The peroxisome proliferator-activated receptors (PPARs) are ligand-triggered transcription factors. Upon ligand binding, they act on DNA response elements (PPREs) in the promoters of target genes as heterodimers with the retinoid X receptor (RXR), causing gene transcription modulation [11]. The expression of PPARα is significant in the liver and tissues of high metabolic rate [12]. Staels et al. [13] stated that, in smooth muscle cells, PPARα activators showed anti-inflammatory activities by interfering adversely with the nuclear factor-kB (NF-κB) signaling pathway. Furthermore, PPARα negatively interacts with the transcription factor AP-1 [14]. Recently, hepatotoxicity has been proven to involve PPARα inhibition [15], while the hepatoprotective effect of natural compounds has been achieved by targeting PPARα, as well as diminishing oxidative stress [16]. Therefore, there is a need for economical and safe natural antioxidant products used as therapeutic agents for treating GA3-induced hepatotoxicity via decreasing oxidative stress and inflammation, and up-regulating PPARα.

Royal jelly (RJ) is a white viscous milky fluid secreted from the hypopharyngeal gland of worker honey bees (*Apis mellifera* Linne). It contains high levels of amino acids, proteins, lipids, sugars, vitamins, and minerals [17]. Due to its important biological properties, RJ is used as a dietary supplement and in various industries, such as pharmaceuticals, food, and cosmetics [18]. RJ exhibits anti-inflammatory, antioxidant, anti-tumor, immunomodulatory [19], cytoprotective [20], and hepatoprotective [21] activities, as well as triggers hepatocyte regeneration and development [22].

*Chlorella vulgaris* (CV), a unicellular green alga that grows in freshwater, is one of the food supplements widely used around the world [23]. It is documented as a safe alga by the FDA [24]. It contains bioactive compounds such as pigments, proteins, vitamins, and other growth factors [25]. The high content of carotenoids and other bioactive components has shown anti-inflammatory, immunity-modulating, and anti-cancer properties [26]; in addition to hepatoprotective and antioxidative properties [27]. 

Therefore, the present work was performed to estimate the probable ameliorating effects of RJ and CV on gibberellic acid-induced chromosomal alterations in bone marrow cells, as well as biochemical, histopathological, immunohistochemical, and molecular changes involved in gibberellic acid-produced liver toxicity in rats. 

## 2. Materials and Methods

### 2.1. Chemicals

Gibberellic acid (99% purity) as white crystalline powder was supplied by Sigma-Aldrich (Saint Louis MO, USA). Royal jelly soft gelatin capsules were supplied from Pharco pharmaceuticals (Alexandria, Egypt). *Chlorella vulgaris* powder was provided by Algal Biotechnology Unit (National Research Centre, Dokki, Giza, Egypt). Kits determining serum alanine aminotransferase (ALT), aspartate aminotransferase (AST), and alkaline phosphatase (ALP) were obtained from Biosystems (Spain). Gamma-glutamyl transferase (γGT) and total bilirubin measuring kits were bought from Spinreact (Girona, Spain). The serum albumin level was measured using a kit from Bio-Med (Germany). Kits measuring malondialdehyde (MDA), superoxide dismutase (SOD), catalase (CAT), and glutathione peroxidase (GPx) were supplied from Biodiagnostic (Giza, Egypt). ELISA kits for NF-κB and TNF-α were obtained from ELISAGenie (Dublin, Ireland) and Ray Biotech (Norcross, GA, USA), respectively. Other substances had high analytical grades and were provided by suitable sources.

### 2.2. Experimental Animals

Seventy-two healthy male albino rats (120–140 g) were purchased from El-Giza National Research Center (Dokki, Egypt). All procedures dealing with the rats followed the approval of the Institutional Research Ethics Committee of Beni-Suef University (BSU-IACUC, Approval No. 021-180). The rats were housed in well-aerated cages under normal laboratory conditions at 12 h light and dark cycle and 25 ± 2 °C. Animals freely accessed water and standard rat chow diets. 

### 2.3. Experimental Plan and Sampling

After adaptation for one week, the animals were indiscriminately allocated into six groups with 12 rats/each, divided into 2 separate cages, and treated orally using gastric gavage once daily for 6 weeks. In each group, six rats were used for cytogenetic analysis. The others were used for investigating hepatotoxicity.

Group I: Negative control rats were provided with distilled water through oral intubation.

Group II (RJ): Rats were orally administered 300 mg/kg/day royal jelly, suspended in distilled water [28].

Group III (CV): Rats were orally administered 500 mg/kg/day *Chlorella vulgaris*, suspended in distilled water [29].

Group IV (GA3): Rats were orally administered 55 mg/kg/day gibberellic acid, suspended in distilled water [30].

Group V (GA3 + RJ): Rats received 55 mg/kg GA3, followed by a dose of 300 mg/kg royal jelly.

Group VI (GA3 + CV): Rats received 55 mg/kg GA3, followed by a dose of 500 mg/kg *Chlorella vulgaris*.

At the experiment end, blood samples from 6 animals per group were gathered from the retro-orbital venous plexus and left to coagulate at room temperature. After centrifugation at 3000 rpm for 15 min, sera were collected and conserved at −20 °C until use. Cervical dislocation under mild anesthesia was applied, and then livers were removed and washed with cold saline. Each liver specimen was divided into 3 parts. The first part was used for histopathological and immunohistochemical examination (fixed in 10% neutral buffered formalin). The second part was kept at −70 °C until assessing gene expression. The third part was used for preparing tissue homogenate (10% *w*/*v*) by cold phosphate-buffered saline (10X, pH 7.4), then centrifuged by high-speed cooling centrifuge for 10 min at 3000 rpm, and the obtained clear supernatants were kept at −20 °C. 

### 2.4. Cytogenetic Assay

The colchicine hypotonic procedure was used to prepare bone marrow cells for chromosomal abnormalities and mitotic index analyses. From each group, six animals were sacrificed by cervical dislocation. Two hours before sacrifice, 4 mg/kg colchicine was given intraperitoneally; then, the smears of bone marrow from animals in each group were prepared according to Preston et al. [31]. Slides were stained with Giemsa staining and 50 well-spread metaphase/animal were examined for chromosomal abnormalities. The mitotic index was determined as the dividing cells number/1000 cells/animal.

### 2.5. Estimation of Biochemical Assays

#### 2.5.1. Assay of Liver Function Biomarkers

ALT and AST levels were measured in the serum spectrophotometrically at 340 nm using a Hitachi spectrophotometry, Model U-2000 (Hitachi Ltd., Tokyo, Japan) by using reagent kits purchased from Biosystems, Spain (Cat. No. 11832 & 11830, respectively) as described by IFCC reference procedures [32]. Serum ALP (Biosystems, Barcelona, Spain; Cat. No. 11590) and γGT (Spinreact, Girona, Spain; MD 41288) activities were measured spectrophotometrically at 405 nm according to Tietz [33] and Young [34], respectively. Serum albumin was measured according to Doumas and Biggs [35] at wavelength 623 nm using kits purchased from BioMed (Hannover, Germany; ALB100100). Total bilirubin was determined spectrophotometrically according to David and Michael [36] at 546 nm by Spinreact (Girona, Spain; MD1001042) kits.

#### 2.5.2. Assay of Oxidant/Antioxidant Indices

Lipid peroxidation, as the malondialdehyde level, was assayed spectrophotometrically (Hitachi spectrophotometry, Tokyo, Japan) at 534 nm in the liver homogenate using Biodiagnostic kits (Giza, Egypt, Cat. No. MD 2529) in agreement with the method of Ohkawa et al. [37]. Using Biodiagnostic kits (Giza, Egypt), CAT (Cat. No. CA2517, at 510 nm), SOD (Cat. No. SD2521, at 560 nm), and GPx (Cat. No. GP2524, at 340 nm) were determined spectrophotometrically (Hitachi spectrophotometry, Tokyo, Japan) following the methods of Aebi [38], Nishikimi et al. [39], and Paglia and Valentine [40], respectively.

#### 2.5.3. Assay of Serum TNF-α and NF-κB Levels

Following the manufacturer’s guidelines, serum levels of TNF-α and NF-κB were measured using ELISA kits from Ray Biotech (Norcross, GA, USA; ELM-TNFa) and ELISAGenie (Dublin, Ireland, RTFI00988), respectively. The optical density (OD) was measured spectrophotometrically at 450 nm using Hitachi spectrophotometry (Tokyo, Japan).

### 2.6. Quantitative Real Time-Polymerase Chain Reaction (qRT-PCR) for Detection of PPARα and AP-1 Genes Expression Level

The gene expression level of PPARα and AP-1 in liver tissue of all experimental groups was performed by qRT-PCR. Total RNA was extracted by total RNA isolation kits (Thermo Scientific, Waltham, MA, USA) and quantified at 260 nm. RNA samples of 1.8 and higher A260/A280 were chosen for reverse transcription to form cDNA using a RevertAid First Strand cDNA Synthesis Kit (Thermo Scientific, Waltham, MA, USA). cDNA was amplified by SYBR Green master mix (Fermentas, Waltham, MA, USA) using the primer set (described in Table 1) in a total volume of 20 μL. The acquired amplification data were analyzed by the 2^−ΔΔCt^ method [41] and the values were standardized to β-actin.

### 2.7. Histopathological Assessment

#### 2.7.1. Microscopic Evaluation

After the experiment completion, liver specimens from all experimental groups were directly immersed in 10% formalin fixative for two days. Thereafter, they were transacted to the usual paraffin technique and the next stains as explained by Suvarna et al. [42].

a. General histological analysis:
  1. Haematoxylin and eosin (H&E) for general screening and semi-quantitative scoring of the hepatic damages in 6 fields X200 from each group. The hepatic alterations are cellular vacuolation, apoptosis, vascular congestion, and inflammation (inflammatory cell infiltrations). Scores were estimated as follows: 0 = no changes, 1 = alterations are <25%, 2 = alterations are 26–50%, 3 = alterations are 51–75%, and 4 = alterations are 75% of studied fields under the light microscope as measured by Gibson-Corley [43].
  2. Crossmon’s trichrome for clarification of collagen fibers and their area percentage.
b. Histochemical analysis:

1. Periodic acid-Schiff (PAS) stain for detection of cellular mucopolysaccharides secretions and their area percentage.
2. Bromophenol blue stain for detection of cytoplasmic total protein contents and their area percentage.

c. Immunohistochemical analysis: Applied on the paraffinized liver sections of all studied groups mounted on positive slides as follows:

1. COX-2 immunohistochemistry: detection of COX-2 expression by using anti-COX-2 rabbit monoclonal antibody [EPR12012] (ab179800) [44]. The routine technique is performed till washing with water. Then, antigen retrieval was done by immersion of sections in Tris/EDTA buffer (10 mM Tris Base, 1 mM EDTA Solution, 0.05% Tween 20, pH 9) for 15 min, heat to 98 °C, and then let cool for 10–20 min. Sections must be flooded with 3% H_2_O_2_ for 5 min in methyl alcohol to suppress the endogenous peroxidase and then washed in TBS (10X, pH 7.4) plus 0.025% Triton X-100 with gentle agitation. At 37 °C, make blocking in 10% normal serum + 1% BSA in TBS for two hrs. Do not rinse and let slides dry. Applying the primary antibody (Anti-COX-2) diluted in TBS + 1% BSA 1:250 presented from Abcam, United States. Overnight incubation at 4 °C then rinse with gentle agitation in TBS 0.025% Triton. Apply the secondary antibody mingled with TBS + 1% BSA, and then incubate at 37 °C for 1 h. Stain sections with a chromogen Anti-COX-2 rabbit monoclonal antibody [EPR12012] (ab179800), at 37 °C for 10 min; then rinse with running tap water for 5 min. Stain the nuclei with Mayer’s hematoxylin for 2 min; then dehydrate, clear, and mount sections.
2. iNOS immunohistochemistry: detection of iNOS expression by using Anti-iNOS rabbit polyclonal antibody (ab15323) [45]. In a water bath, make antigen retrieval by immersion of sections in sodium citrate buffer (10 mM Sodium Citrate, 0.05% Tween 20, pH 6.0) at 95 °C for 20 min, wash with PBS for 5 min, incubate sections with PBS (10X, pH 7.4) containing 0.1–0.25% Triton X-100 for 10 min, wash with PBS for 5 min, then stain with Anti-iNOS primary antibody presented from Abcam, Cambridge, UK, diluted 1:100 with 1% bovine serum albumin (BSA) + phosphate-buffered saline with Tween 20 (PBST) (1% BSA in PBST 1X, pH 7.0) at 4 °C for 12 h in a humidified chamber, rinse by TBS for 5 min, stain sections in the dark with the secondary antibody +1% BSA for 1 h at 37 °C, wash by PBS for 5 min in the dark, rinse with PBS, stain the nuclei with Mayer’s hematoxylin for 2 min, then dehydrate, clear, and mount sections.

#### 2.7.2. Image Analysis

Image analysis was done to gauge the area percentage (6 fields in each group X400) of each of the following:

1. Total collagen fibers in Crossmon’s trichrome-stained liver sections.
2. Glycogen content in PAS-stained sections.
3. Total protein content in Bromophenol blue-stained sections.
4. Positive COX-2 content expression in immunostained sections with COX-2 antibody.
5. Positive iNOS content expression in immunostained sections with iNOS antibody.

A LEICA digital camera (DFC290 HD system, Morrisville, TN, USA) was used to screen and capture all stained hepatic sections. The assessment was completed by a freeware program (Image-J 1.52a).

### 2.8. Statistical Analysis

SPSS (version 25, Chicago, IL, USA) was used to carry out the statistical analysis. Data were represented as mean ± standard deviation (SD). All statistical comparisons were done using a one-way ANOVA test with Tukey’s test post hoc analysis. The value of (*p* ≤ 0.05) was judged significant.

## 3. Results

### 3.1. RJ and CV Decrease Cytogenetic Toxicity Induced by GA3 in Rats

The results obtained from the examination of rat bone marrow cells at the metaphase stage are shown in Table 2. The investigated structural chromosomal aberrations included deletions, breaks, ring chromosomes, fragments, end-to-end association, centromeric attenuation, and centric fusion. While aneuploidy (metaphases with more or less 42 chromosomes) and polyploidy (metaphases with more than 2 haploid sets of chromosomes) were examined to depict the numerical chromosomal aberrations. GA3 provoked a meaningful (*p* ≤ 0.001) increase in total structural and numerical chromosomal aberrations. Deletion, break, and ring chromosome were the most observed structural chromosomal aberrations and considerably (*p* ≤ 0.001) increased relative to the control group. End-to-end association (*p* ≤ 0.001), centric fusion (*p* ≤ 0.001), centromeric attenuation (*p* ≤ 0.01), and fragments (*p* ≤ 0.05) were also raised significantly over the control group. 

Aneuploidy as the most frequent numerical chromosomal aberration along with polyploidy were significantly increased in GA3-administered rats at (*p* ≤ 0.001) and (*p* ≤ 0.05), respectively, in comparison with the control rats.

Treatment of GA3-intoxicated rats with RJ and CV significantly decreased most of the detected types of structural and numerical chromosomal aberrations. Additionally, both the total number of structural and numerical chromosomal aberrations were considerably decreased (*p* ≤ 0.001) in GA3-administered rats after treatment with either RJ or CV.

**Figure 1:**
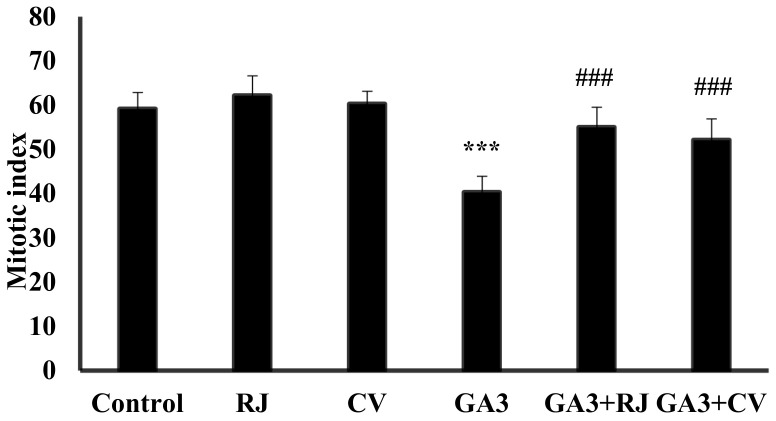
Effect of RJ and CV on the mitotic index of control and GA3-induced rats. The mitotic index was obtained by counting 6000 mitotic cells per treatment. Data are expressed as Mean ± SD (N = 6). *** *p* ≤ 0.001 vs. Control, ^###^ *p* ≤ 0.001 vs. GA3.

In contrast, the mitotic index (assessed by the ratio of cells undergoing mitosis to those of non-dividing cells) was significantly decreased (*p* ≤ 0.001) in the GA3-induced group compared with the control group indicating bone marrow cytogenetic toxicity (Figure 1). While the mitotic index in the groups treated with RJ and CV concurrently with GA3 was increased considerably (*p* ≤ 0.001) indicating anti-cytogenotoxicity towards GA3, as shown in Figure 1.

**Table 2:** Effect of royal jelly (RJ) and *Chlorella vulgaris* (CV) on chromosomal aberrations of bone marrow cells in control and gibberellic acid (GA3)-induced rats.

Groups	Structural Chromosomal Aberrations								Numerical Chromosomal Aberrations			TCA
	Chromatid Break	Chromatid Deletion	Ring Chromosome	Fragment	End to End Association	Centric Fusion	Centromeric Attenuation	TSA	Polyploidy	Aneuploidy	TNA	
Control	1 ± 1.3	0.7 ± 0.5	0.5 ± 0.8	0.5 ± 0.5	0.0 ± 0.0	0.0 ± 0.0	0.0 ± 0.0	2.7 ± 0.8	0.5 ± 0.5	0.3 ± 0.8	0.8 ± 1.2	3.5 ± 1
RJ	0.8 ± 1	0.5 ± 0.5	0.3 ± 0.4	0.3 ± 0.5	0.0 ± 0.0	0.0 ± 0.0	0.0 ± 0.0	2 ± 1	0.3 ± 0.5	0.2 ± 0.4	0.5 ± 0.5	2.5 ± 1
CV	1 ± 0. 9	0.7 ± 0.8	0.3 ± 0.5	0.5 ± 0.8	0.0 ± 0.0	0.0 ± 0.0	0.0 ± 0.0	2.5 ± 1	0.5 ± 0.8	0.3 ± 0.5	0.8 ± 0.8	3.3 ± 0.8
GA3	5.5 ± 1 ***	6.7 ± 1 ***	4.7 ± 1 ***	2 ± 1.3 *	1.5 ± 1***	1.7 ± 0.8 ***	1.3 ± 1.2 **	23 ± 1 ***	2 ± 1.3 *	2.8 ± 1.5 ***	4.8 ± 1.2 ***	28 ± 0.8 ***
GA3 + RJ	2.3 ± 1.2 ^###^	2.7 ± 1.2 **^###^	1.3 ± 1.2 ^###^	0.5 ± 0.5 ^#^	0.3 ± 0.5 ^##^	0.5 ± 0.8 ^##^	0.2 ± 0.4 ^#^	7.8 ± 1.2 ***^###^	0.5 ± 0.5 ^#^	0.8 ± 1 ^##^	1.3 ± 0.8 ^###^	9.2 ± 1.5 ***^###^
GA3 + CV	2.7 ± 1.4 ^##^	3.2 ± 1.2 ***^###^	1.8 ± 1.5 ^###^	0.7 ± 0.8	0.5 ± 0.5 ^#^	0.5 ± 0.5 ^##^	0.3 ± 0.5 ^#^	9.7 ± 2 ***^###^	0.7 ± 0.5 ^#^	1.2 ± 1 ^#^	1.8 ± 1 ^###^	12 ± 2 ***^###^

50 cells were analyzed per animal for chromosomal aberrations. Six animals were examined per group. Data are expressed as Mean ± SD (N = 6). TSA: Total Structural Aberrations; TNA: Total Numerical Aberrations; TCA: Total Chromosomal Aberrations. * *p* ≤ 0.05; ** *p* ≤ 0.01, *** *p* ≤ 0.001 versus (vs) Control. ^#^ *p* ≤ 0.05, ^##^ *p* ≤ 0.01, ^###^ *p* ≤ 0.001 vs. GA3.

### 3.2. RJ and CV Ameliorate Liver Function Biomarker Changes Induced by GA3 in Rats

Data represented in Table 3 revealed that the GA3-induced group had significantly (*p* ≤ 0.001) higher levels of ALT, AST, ALP, γGT, and total bilirubin, as well as significantly (*p* ≤ 0.001) lower levels of albumin. Rats that received RJ and CV simultaneously with GA3 showed a significant (*p* ≤ 0.001) amelioration of ALT, AST, ALP, γGT, and total bilirubin. Albumin was meaningfully raised in RJ (*p* ≤ 0.001) and CV (*p* ≤ 0.01) treated groups compared to GA3 intoxicated rats.

### 3.3. RJ and CV Alleviate Hepatic Oxidative Stress Induced by GA3 in Rats

GA3-intoxicated rats exhibited a status of oxidative stress as manifested by a significant (*p* ≤ 0.001) elevation in hepatic malondialdehyde level accompanied by a significant (*p* ≤ 0.001) reduction in SOD, CAT, and GPx activity (Table 4). The group of rats treated with royal jelly simultaneously with gibberellic acid showed a remarkable (*p* ≤ 0.001) decrease in MDA content and increase in SOD, CAT, and GPx activity. Similarly, treatment of GA3-intoxicated rats with *Chlorella vulgaris* significantly (*p* ≤ 0.001) suppressed lipid peroxidation, and significantly (*p* ≤ 0.001) enhanced activity of SOD, CAT, and GPx enzymes (Table 4).

### 3.4. RJ and CV Mitigate Inflammation Induced by GA3 in Rats

**Figure 2:**
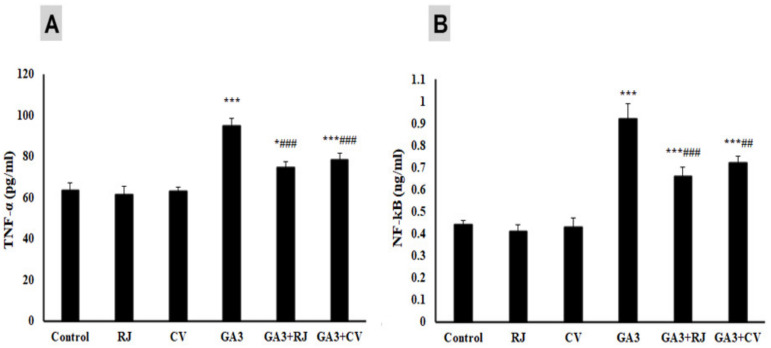
Effect of RJ and CV on tumor necrosis factor-alpha (TNF-α) (**A**) and nuclear factor-κB (NF-κB) (**B**) in control and GA3- induced rats. Data are expressed as Mean ± SD (N = 6). * *p* ≤ 0.05, *** *p* ≤ 0.001 vs. Control, ^##^ *p* ≤ 0.01, ^###^ *p* ≤ 0.001 vs. GA3.

The anti-inflammatory effect of RJ and CV against GA3- triggered inflammation was assessed by measuring TNF-α (Figure 2A) and NF-κB (Figure 2B) serum levels. Circulating levels of TNF-α were markedly (*p* ≤ 0.001) increased in the GA3-administered group as compared to the control group. RJ or CV supplementation revealed a significant (*p* ≤ 0.001) reduction in serum levels of TNF-α compared with the GA3-administered group. Likewise, the GA3-administered group showed a significantly (*p* ≤ 0.001) elevated NF-κB level, an effect that was significantly reduced after the administration of RJ (*p* ≤ 0.001) or CV (*p* ≤ 0.01). 

### 3.5. RJ and CV Up-Regulate PPAR Alpha and Down-Regulate AP-1 in the Liver of GA3-Intoxicated Rats

**Figure 3:**
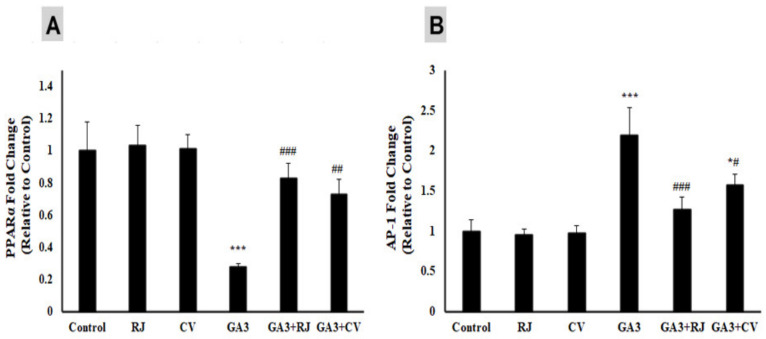
Effect of RJ and CV on gene expression level of PPARα (**A**) and AP-1 (**B**) in liver of control and GA3- induced rats. PPARα: Peroxisome proliferator activated receptor α; AP-1: activator protein 1. * *p* ≤ 0.05, *** *p* ≤ 0.001 vs. Control. ^#^ *p* ≤ 0.05, ^##^ *p* ≤ 0.01, ^###^ *p* ≤ 0.001 vs. GA3.

PPARα mRNA abundance (Figure 3A) was notably (*p* ≤ 0.001) decreased in the liver of GA3-intoxicated rats as compared to the control rats. RJ (*p* ≤ 0.001) and CV (*p* ≤ 0.01) administration up-regulated the expression of PPARα when compared with the GA3-intoxicated group.

In contrast, AP-1 mRNA in the liver of GA3-intoxicated rats underwent a significant up-regulation (*p* ≤ 0.001) in comparison with the corresponding control group as represented in Figure 3B. Oral administration of either RJ (*p* ≤ 0.001) or CV (*p* ≤ 0.05) significantly down-regulated the AP-1 gene expression level in comparison with the GA3-intoxicated rats.

### 3.6. RJ and CV Attenuate Tissue Injury and Fibrosis in the Liver of GA3-Intoxicated Rats

**Figure 4:**
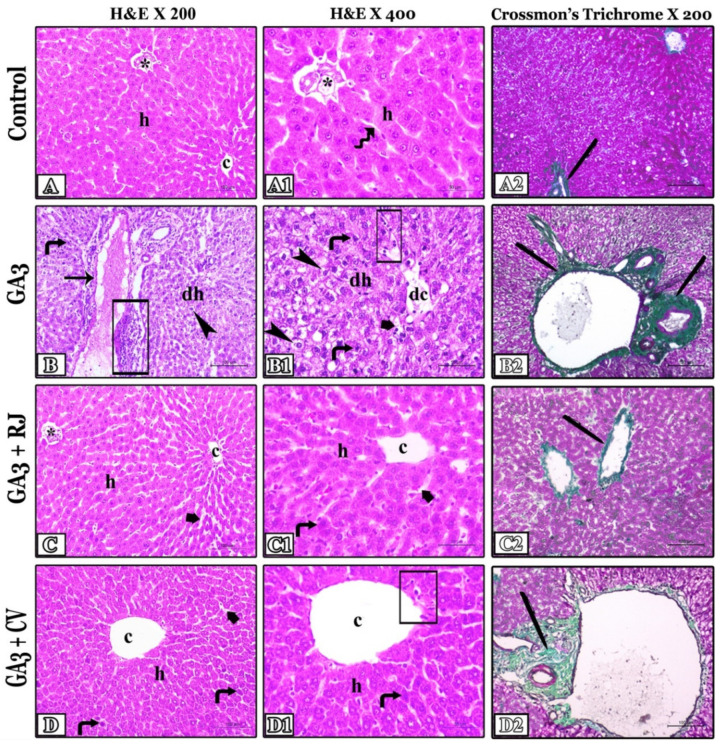
Representative photomicrographs of liver cross sections in all studied groups: 1st and 2nd columns stained with H&E (X200 and X400, respectively); Control rats (**A**,**A1**) showing typical hepatic cords of acidophilic polygonal hepatocytes with vesicular nuclei (h) appeared as rays from the central vein (c) separated by typical blood sinusoids (zigzagged arrow) and normal portal areas (*). GA3-treated group (**B**,**B1**) showed degenerated hepatocytes (dh), numerous vacuolated cells (arrowheads), numerous apoptotic cells (turned arrows), degenerated central veins (dc), degenerated sinusoids (short thick arrows), dilated lymphatic vessels with disrupted lining (long arrow), periportal and perivascular lymphocytic infiltration (rectangle). GA3 + RJ (**C**,**C1**) and GA3 + CV (**D**,**D1**)-treated groups exhibited an improvement of all alterations and showed normal hepatocytes (h), normal central vein (c), portal area (*), few apoptotic cells (turned arrows), few degenerated sinusoids (short thick arrows), and intercellular lymphocytic infiltration (rectangle). 3rd column was stained with Crossmon’s trichrome stain X 200; the control group (**A2**) showed normal fine interlobular and perivascular collagen fibers of green color (pens). GA3-treated group (**B2**) has a massive proliferation of collagen fibers surrounding the degenerated vessels. GA3 + RJ-treated group (**C2**) showed normal distribution of collagen fibers compared to GA3-treated group. GA3 + CV-treated group (**D2**) showed reduction in the proliferated fibers that appeared only as mild perivascular proliferation. Pens indicate green-colored collagen fibers.

H&E stained liver sections from the control, RJ, and CV treated groups showed a normal hepatic structure. There were undeveloped connective tissue septa separating the classic lobules, which were formed of typical hepatic cords of polygonal hepatocytes that appeared as rays from the central vein containing cytoplasm with acidophilia and round centric nuclei, as well as typical peripheral portal areas. The radiating cords were separated by blood sinusoids with normal lining (Figure 4(A,A1)). On the contrary, the GA3-treated group showed severe irregularity and degeneration of the hepatocytes, besides inflammation. Numerous cells exhibited cytoplasmic hydropic degeneration (vacuolated cytoplasm) and nuclear degeneration, and others appeared apoptotic with condensed nuclei. Cells surrounding portal areas were more degenerated than those surrounding central veins. Most of the central veins, sinusoids, and portal vessels including lymphatic vessels appeared dilated with disrupted lining, congested, and containing tissue exudate with lymphocytic infiltration. Multifocal areas of lymphocytic infiltrations appeared surrounding the portal areas and central veins (Figure 4(B,B1)). All previously mentioned alterations caused by GA3 were ameliorated with the administration of RJ. Most of the cells were improved and returned to their normal structure but a few sinusoids were still dilated with degenerated lining, as well a few apoptotic cells were present (Figure 4(C,C1)). Also, in the group treated with CV, most of the pathological changes were partially alleviated, but mild changes in some hepatocytes and some apoptotic cells were still observed. Additionally, a few focal areas of periportal lymphocytic infiltrations and around the central veins with vascular dilatation were detected (Figure 4(D,D1)). The scoring of pathological alterations in all experimental groups is presented in Table 5.

In hepatic slides stained with Crossmon’s trichrome, collagen fibers and their area percentage were detected and calculated. The control group showed normal distribution of interlobular and perivascular fine collagen fibers of green color (Figure 4A2). On the contrary, the administration of GA3 led to the initiation of liver fibrosis, in that the degenerated vessels were surrounded by excessive proliferation of fibers in comparison with the normal control (Figure 4B2). In the GA3 + RJ-treated group, fibers appeared with normal distribution compared with the GA3-treated group (Figure 4C2). Similarly, the proliferation of fibers was diminished by the treatment using CV, and there was only mild proliferation of perivascular fibers (Figure 4D2). The area quantity calculation of the collagen fibers in all experimental groups is shown in Table 6. It ensured that GA3 leads to the induction of fibrosis. A significant increase in fiber quantity appeared in the GA3-treated group (*p* ≤ 0.001) in comparison with normal. In contrast, a significant decrease in fibers (*p* ≤ 0.001) appeared in GA3 + RJ and GA3 + CV-treated groups in comparison with the GA3-treated group.

**Table 5:** The scores of histopathological alterations in liver sections of all studied groups.

Groups	Vascular Congestion	Apoptosis	Cellular Vacuolation	Inflammation (Inflammatory Cell Infiltrations)
Control	0.0 ± 0.0	0.0 ± 0.0	0.0 ± 0.0	0.0 ± 0.0
RJ	0.3 ± 0.6	0.3 ± 0.3	0.0 ± 0.0	0.0 ± 0.0
CV	0.7 ± 0.6	0.3 ± 0.3	0.0 ± 0.0	0.0 ± 0.0
GA3	3.7 ± 0.6 ***	3.3 ± 0.6 ***	3.7 ± 0.6 ***	3.7 ± 0.6 ***
GA3 + RJ	0.7 ± 0.6 ^###^	0.7 ± 0.6 ^###^	0.7 ± 0.0 **^###^	0.7 ± 0.6 ^###^
GA3 + CV	1.3 ± 0.6 **^###^	1.3 ± 0.6 **^###^	1.3 ± 0.6 ***^###^	1.3 ± 0.6 **^###^

Data are expressed as Mean ± SD (N = 6). ** *p* ≤ 0.01, *** *p* ≤ 0.001 vs. Control. ^###^ *p* ≤ 0.001 vs. GA3.

**Table 6:** Comparative analysis of collagen fibers, PAS, Bromophenol blue, COX-2, and iNOS area percentages in all experimental groups.

Groups	Area Percentage of				
	Collagen Fibers	PAS-Positive Content	Bromophenol Blue-Positive Content	COX-2 Immuno- Expression	iNOS Immuno- Expression
Control	1.3 ± 0.1	75.4 ± 1.4	77 ± 1	0.6 ± 0.1	0.13 ± 0.04
RJ	1.7 ± 0.2	74.3 ± 1.3	75 ± 1	0.6 ± 0.2	0.15 ± 0.03
CV	1.6 ± 0.3	73.4 ± 1.5	75 ± 1	0.7 ± 0.1	0.2 ± 0.1
GA3	28 ± 2 ***	23 ± 2 ***	14 ± 1 ***	70 ± 1.5 ***	5.2 ± 1.6 ***
GA3 + RJ	3.2 ± 0.5 **^###^	71.5 ± 1.1 **^###^	68.4 ± 1.1 ***^###^	3.9 ± 0.7 **^###^	0.7 ± 0.1 ^###^
GA3 + CV	7.6 ± 0.8 ***^###^	68 ± 2 ***^###^	62 ± 2 ***^###^	28.5 ± 2.4 ***^###^	2.2 ± 0.6 **^###^

Data are expressed as Mean ± SD (N = 6). PAS: Periodic acid-Schiff; COX-2: cyclooxygenase 2; iNOS: inducible nitric oxide synthase. ** *p* ≤ 0.01, *** *p* ≤ 0.001 vs. Control. ^###^ *p* ≤ 0.001 vs. GA3.

### 3.7. RJ and CV Attenuate Histochemical Changes in the Liver of GA3-Intoxicated Rats 

**Figure 5:**
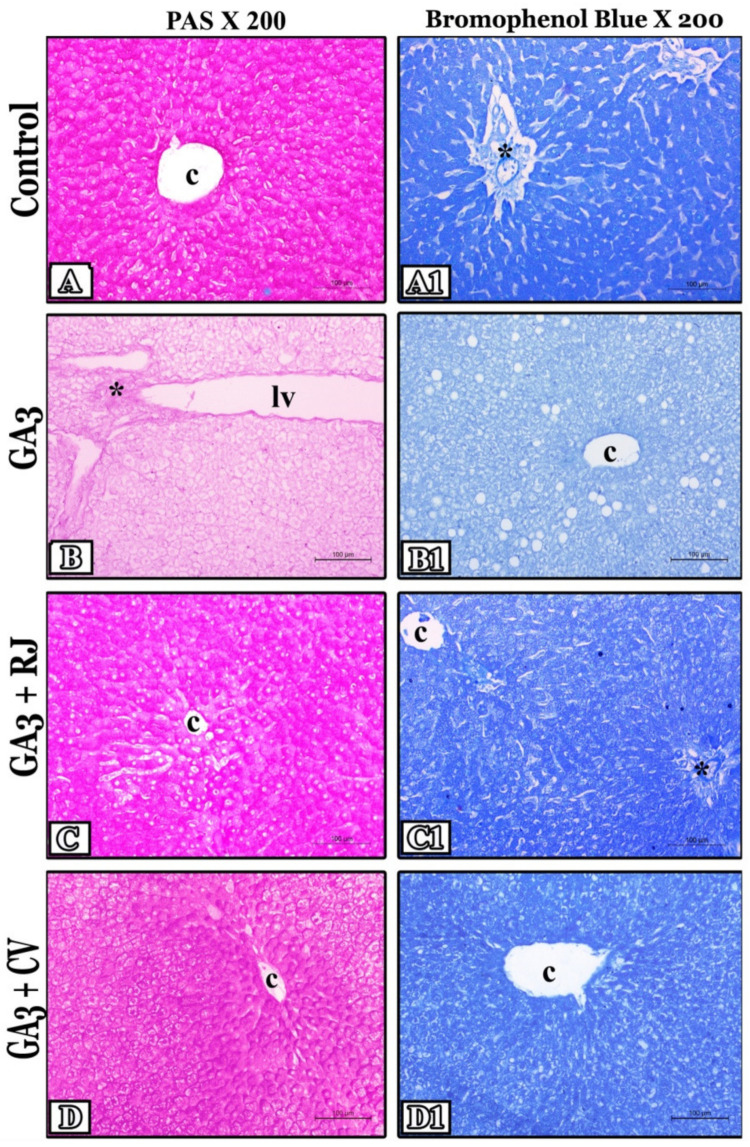
Histochemical assessment of liver in all studied groups by Periodic acid-Schiff (PAS)-stained sections X 200 (1st column) and Bromophenol blue-stained sections X200 (2nd column). The control group showed normal distribution of cytoplasmic glycogen (**A**) and total protein contents (**A1**) exhibited by strong positive colors (magenta and blue, respectively). The GA3-treated group showed great depletion of glycogen (**B**) and protein contents (**B1**) so appeared with faint colors. GA3 + RJ (**C**,**C1**) and GA3 + CV-treated groups (**D**,**D1**) showed improved production of both secretions, and thus appeared with moderate to strong colors. Notice: central vein (c), portal area (*) and lymphatic vessel (lv).

In PAS-stained liver sections, the distribution of cytoplasmic glycogen appeared with a typical strong positive magenta red reaction in the control group (Figure 5A). On the contrary, the GA3-treated group showed a great depletion of glycogen content, which was indicated by very faint coloration as compared with the control (Figure 5B). While, after administration of RJ, the secretion of glycogen was returned to normal after appearing in the form of a strong PAS color when compared with the GA3-treated group (Figure 5C). Also, glycogen content was partially restored in the GA3 + CV-treated group, which was indicated by a strong reaction in the normal hepatocytes and a moderate one in the others (Figure 5D). Image analysis of the area percentage of PAS-positive content was recorded in all studied groups in Table 6. A significant decrease (*p* ≤ 0.001) was detected in the GA3-treated group compared with the control group. Conversely, in GA3 + RJ and GA3 + CV-treated groups, there was a significant (*p* ≤ 0.001) increase in comparison with the GA3-treated group. 

Regarding the Bromophenol blue stain, hepatic sections of the control showed typical cytoplasmic protein content that appeared as a strong dark blue coloration (Figure 5A1). On the contrary, a marked reduction of protein appeared in the GA3-treated group manifested by a faint blue reaction compared with the normal (Figure 5B1). While, in the group treated with RJ, the previous depletion of protein was alleviated and the secretion was restored to normal, which was manifested by a strong blue color in comparison with the GA3-treated group (Figure 5C1). Also, the GA3 + CV-treated group revealed partial improvement in protein secretion indicated by a strong reaction in the normal hepatocytes and a moderate one in the others (Figure 5D1). Image analysis of the area percentage of the Bromophenol blue positive reaction was recorded in Table 6. In the GA3-treated group, a significant decrease (*p* ≤ 0.001) was showed in comparison with control. But, a significant increase (*p* ≤ 0.001) was revealed in GA3 + RJ and GA3 + CV-treated groups compared to the GA3-treated group. 

### 3.8. RJ and CV Downregulate COX2 and iNOS Immunoexpression in the Liver of GA3-Intoxicated Rats

**Figure 6:**
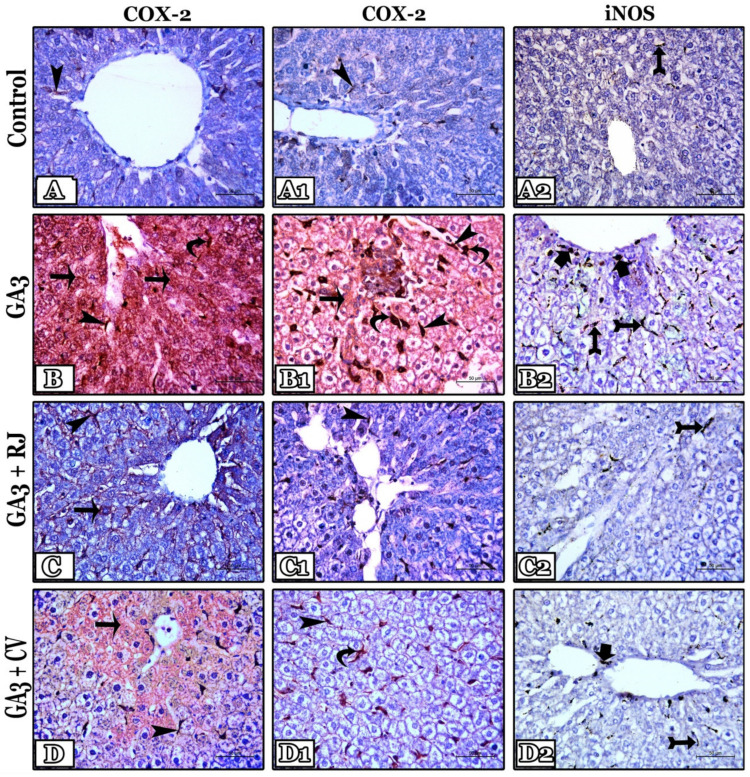
Representative photomicrographs of cyclooxygenase 2 (COX-2) immunoexpression in liver sections of all studied groups (1st column showing the tissue in the central area and 2nd column showing the tissue in the portal area (X400)): Control group (**A**,**A1**) showed few positive, brown-colored COX-2 granules in the sinusoidal endothelium near the central vein. GA3-treated group (**B**,**B1**) revealed a marked raise of the positive granules with high intensity in all hepatocytes, the sinusoidal lining, and the perivascular tissue. GA3 + RJ group (**C**,**C1**) showed few positive granules expressed only in few hepatocytes and the sinusoidal endothelium compared to the GA3-treated group. The GA3 + CV group (**D**,**D1**) showed mild COX-2 expression of low intensity in some hepatocytes and the sinusoidal lining around the central vein. Notice, the positive COX-2 granules in hepatocytes (thin long arrows), sinusoidal endothelium (arrowheads), and Van Kupffer cells (curved arrows). Immuno-stained inducible nitric oxide synthase (iNOS) liver sections of all studied groups (3rd column X400): Control group (**A2**) showed fine positive dark brown-colored iNOS granules in the sinusoidal endothelium near the central vein. The GA3-treated group (**B2**) showed numerous positive granules in the sinusoidal endothelium of the periportal area and the perivascular tissue in comparison to the control. GA3 + RJ (**C2**) showed positive iNOS granules only in few sinusoidal endothelium. GA3 + CV (**D2**) showed positive granules in some sinusoidal lining and the perivascular tissue in the periportal area. Notice, the positive iNOS granules in the sinusoidal endothelium (tailed arrows) and perivascular tissue (thick short arrows).

COX-2-immunostained sections: the control group normally has very few positive, brown granules in the sinusoidal endothelium near the central vein (Figure 6(A,A1)). On the contrary, COX-2 expression was markedly elevated in the GA3-treated group in comparison with the control. It presents as excessive positive granules with high intensity filling numerous hepatocytes, the sinusoidal lining (endothelium and Van Kupfer cells), and in the perivascular tissue mainly around the central vein (Figure 6(B,B1)). Treatment with RJ markedly decreases COX-2 production, so GA3 + RJ group showed few positive contents that were expressed only in few hepatocytes and the sinusoidal endothelium compared to GA3-treated group (Figure 6(C,C1)). Also, administration of CV partially decreases the COX-2 positive contents and thus appeared with a mild intensity only in some hepatocytes mainly around the central vein and in the sinusoidal lining (Figure 6(D,D1)). The area percentage of COX-2 immunoexpression in all studied groups was recorded in Table 6. A significant increase (*p* ≤ 0.001) appeared in GA3-induced group in comparison with control. In contrast, the expression decreased significantly (*p* ≤ 0.001) in GA3 + RJ and GA3 + CV-administered groups in comparison with GA3-induced group.

iNOS immunostained sections: the control group showed fine positive dark brown-colored iNOS granules in the sinusoidal endothelium near the central vein (Figure 6A2). In contrast, iNOS expression was increased markedly in the GA3-treated group; numerous positive granules appeared in the sinusoidal endothelium mainly in the periportal area and the perivascular tissue in comparison to the control (Figure 6B2). While in GA3 + RJ group, iNOS expression markedly decreased and thus appeared only in few sinusoidal endothelium (Figure 6C2). Likewise, the treatment with CV partially decreased iNOS expression; it presents only in some sinusoidal lining and the periportal area (Figure 6D2). The area percentage of iNOS immunoexpression was recorded in Table 6. It was increased significantly (*p* ≤ 0.001) in the GA3-treated group in comparison with the control. In contrast, a significant decrease (*p* ≤ 0.001) was detected in the GA3 + RJ and GA3 + CV-treated groups compared to the GA3-treated group.

### 3.9. Image Analysis and Statistical Evaluation

The comparative analysis and quantification of the area percentages of collagen fibers, PAS, bromophenol blue, COX-2, and iNOS expressions in all studied groups was shown in Table 6. It concluded that the reduction of collagen, COX-2, and iNOS, and the raising of glycogen and total protein area percentages in GA3 + RJ and GA3 + CV-treated groups in comparison with the GA3-treated group approve the protective effect of RJ and CV on the hepatic tissue against injury and inflammation produced by GA3.

## 4. Discussion

The utilization of plant-growth hormones and their effects on health are a matter of concern. Gibberellic acid (GA3), a plant growth regulator, is commonly used in Egypt in agriculture to hasten the growth of vegetables and fruits [46]. Human beings and animals can be exposed to residues of GA3 through consuming GA3-treated plants or drinking contaminated water [47]. As well, agricultural workers dermally contact with GA3 or inhale its powder, causing acute toxicity [48]. The precise mechanism causing its toxicity has not yet been entirely understood. In the current study, the genotoxic effect of GA3 was manifested by the induction of chromosomal aberrations and change in the mitotic index in bone marrow cells of rats. Also, we inspected GA3-induced hepatotoxicity with a focus on oxidative stress, inflammation, and the PPARα/Ap-1 signaling pathway, as well as we tested the protective effects of royal jelly and *Chlorella vulgaris* against GA3- induced toxicity. 

GA3 administration increased the structural and numerical chromosomal aberrations in agreement with preceding studies in human lymphocyte cultures [49], and bone marrow cells of mice [50], rats [51], and rabbits [7]. As a result of GA3 interaction with DNA, chromosomes or chromatids’ terminal ends might be deleted, which make them unstable and create end-to-end associations and ring chromosomes, and may lead to total genomic damage [52]. 

Jovtchev et al. [53] reported that the increase in the incidence of chromosomal aberrations in rat bone marrow cells is attributed to the decrease in the mitotic activity of these cells. This confirms our results, which revealed a substantial reduction in the mitotic index in GA3-induced rats indicating bone marrow cytotoxicity as previously reported by Nassar et al. [51].

Co-treatment of GA3-intoxicated groups with royal jelly or *Chlorella vulgaris* decreased the chromosomal aberrations and increased the mitotic index indicating their anti-cytotoxic activities by following many previous studies. El-Monem [54] previously reported the capacity of royal jelly to defend against the genotoxicity induced by environmental pollutants, which may be attributed to its highly biologically active compounds. Nutrients including lipids, peptides, and proteins play a role in the antioxidant and anti-cancer properties of royal jelly in addition to phenolic and flavonoid components [55]. Furthermore, *Chlorella vulgaris* lowered the cytotoxicity and genotoxicity as demonstrated in an early study due to its content of bioactive compounds and natural antioxidants [56]. Saberbaghi et al. [57] as well showed that *Chlorella vulgaris* is capable of diminishing DNA damage and apoptosis and promoting cell cycle progression due to its antioxidant properties that prevent ROS and free radicals from damaging DNA. Additionally, Makpol et al. [58] confirmed that *Chlorella vulgaris* has a defensive nature and controlled DNA damage generated by H_2_O_2_.

GA3 could exert toxic impacts on numerous soft organs including the liver [59]. It is well documented that the liver is the first organ in toxicological prospects concerning its role in xenobiotics biotransformation, detoxification, and excretion [60]. The results of the present study revealed that GA3 significantly increased the serum levels of AST, ALT, γGT, and ALP hepatic enzymes in line with earlier data described by Wafaa et al. [46]. The normal blood levels of these enzymes result from the continual leaking of minute amounts through the cell membrane within the hepatocytes. However, in the instance of hepatocellular toxicity, the membranes become more porous due to the loss of functional integrity leading to increased serum levels of these enzymes [61]. 

The reduction in the serum albumin as a result of GA3 administration was reported in our results in parallel with Troudi et al. [62]. Albumin is the most abundant blood plasma protein produced in the liver [63]. The declined level points to chronic liver disorders characterized by considerable hepatocyte destruction and deficiency in the synthetic function of the liver [64]. Also GA3 administration was associated with an elevated serum total bilirubin level as previously recorded by Troudi et al. [62]. Bilirubin accretion evaluates the binding, conjugation, and excretion capability of hepatocytes and is one of the best clinical indications of the degree of necrosis. Hence, the significant liver damage was linked to elevated bilirubin levels [65]. 

Nowadays, RJ plays an important role in folk medicine owing to its numerous biological activities [66]. The co-administration of RJ with GA3 exhibited a significant decline in the elevated AST, ALT, γGT, and ALP concentrations and ameliorated the changes of albumin and total bilirubin levels associated with GA3 hepatotoxicity. Our results were in line with Gholie Pour et al. [67] who confirmed that RJ significantly diminished the levels of liver enzymes. The modulating effects of RJ on the liver function enzymes could be attributed to vitamin C, vitamin E, and arginine found in RJ. Vitamins E and C are well-known antioxidants that prevent cell membrane damage caused by free radicals, reduce liver inflammation, and thus reduce enzyme leakage [68].

The current study revealed that CV administration significantly improved liver function biomarkers in harmony with Vakili et al. [69] who explained that CV supplementation showed meaningful improvements in liver enzymes. Non-alcoholic fatty liver diseased patients who consumed CV for three months experienced significant drops in ALT and AST [70]. Another explanation was that CV could protect liver cells by influencing insulin resistance. CV supplementation decreased plasma non-esterified fatty acid concentration improving glucose homeostasis and resulting in a discernible decrease in serum glucose concentrations [71]. Blood glucose levels were correlated with liver enzymes [72].

In the current study, GA3 administration showed a significant increment in MDA level and a significant decrement in SOD, CAT, and GPx activities in hepatic tissues, denoting that GA3 provoked oxidative stress and lipid peroxidation, as illustrated by Hussein et al. [5]. This was related to the generation of hydroxyl radicals, which can react with lipids via hydrogen abstraction and cause lipid peroxidation and oxidative damage inside the cell [73]. Also, ROS can attack thiols in proteins and glutathione causing inactivation of the enzymes [6]. SOD and GSH-Px play a key role in cellular defense against ROS, reducing oxidized lipids and protein targets of ROS [74]. GA3 could down-regulate CAT, SOD, and GPx mRNA in the liver tissues [5]. The diminution in antioxidant enzymes’ activities might be due to the extreme utilization following the flux of superoxide radicals [46].

Interestingly, our results showed that RJ treatment significantly decreased MDA hepatic level and increased the enzymatic activities of SOD, CAT, and GPx. These results were confirmed by You et al. [75] who stated that RJ could mitigate the deleterious effects of oxidative stress by boosting the activity of liver antioxidant enzymes. Kocot et al. [76] mentioned that short-chain peptides, phenolic compounds, and fatty acids are some of the substances obtained from RJ that have been shown to have potent antioxidant properties. Also, aspartic acid, cysteine, and cystine, which are involved in the formation of GSH, a powerful cellular antioxidant, are present in RJ [77]. Khodabandeh et al. [78] clarified that by lowering the leukocyte response and increasing the mitochondrial respiratory chain, RJ contributes to the reduction of lipid peroxidation and production of ROS. So, depending on the best deduction, the antioxidants in RJ have hepatoprotective effects against the harmful effects of free radicals generated by GA3.

Similarly, our results revealed that CV supplementation showed higher SOD, CAT, and GPX activities with significantly lower MDA values in good agreement with Abdel-Tawwab et al. [79]. Phytochemicals; like tocopherols, chlorophylls, flavonoids, carotenoids, ubiquinone, and polyphenols that have antioxidant properties are extensively included in CV [80]. In this concern, Zahran and Risha [81] stated that CV increased CAT and GPX levels in Nile tilapia. Also, *Chlorella* species-derived polysaccharides have demonstrated antioxidant action against free radicals [82]. *Chlorella vulgaris* boosts the body’s overall antioxidant capacity while inhibiting lipid peroxidation to preserve cellular membranes from deterioration [83]. 

The inflammatory reactions associated with GA3 administration were highly obvious in our study in which it increased serum levels of TNF-α and NF-κB in accordance with Soliman et al. [30]. During inflammatory reactions in hepatic tissues, oxidative stress is an imperative factor [84]. Activation of the pro-inflammatory NF-κB pathway via ROS produces TNF-α and other inflammatory mediators [85]. TNF-α plays an important role in the development of liver injury [86], and has been demonstrated to intensify the pathophysiological reactions induced by toxicants [87]. TNF-α induces cell death through apoptotic and necrotic pathways, thus reducing TNF-α production declines in tissue injury [88]. NF-κB, a nuclear transcription factor, controls apoptosis and immunological actions and mediates acute and chronic inflammatory responses [89]. The release of NF-κB from inhibitory protein IκB causes its translocation from the cytoplasm into the nucleus where it binds to the promoters of pro-inflammatory mediators such as TNF-α, IL-1β, and IL-6, resulting in the induction of their gene expression [90]. Cytokines that are stimulated by NF-κB can directly activate the NF-κB pathway, generating a positive autoregulatory loop that can enhance the inflammatory response and frequency of inflammation [91]. 

Administration of RJ to GA3-intoxicated group in the current work significantly diminished the inflammatory mediators induced by GA3. We suggested that the free radicals mediated activation of NF-κB may be alleviated by royal jelly’s antioxidant action in accordance with Almeer et al. [92]. According to Ahmed et al. [17], the management of retinol loss, the antioxidant impact of some free amino acids, and the restoration of ascorbic acid availability by royal jelly are some of the hypothesized explanations for the antioxidant effect. 

Our data confirmed that rats received CV along with GA3 showed a significant reduction in TNF-α and NF-κB circulating levels. Abu-Serie et al. [93] explained that certain phenolics in CV, including gallates, which are powerful TNF-α inhibitors, may be responsible for its anti-inflammatory effect. Additional elements like triterpenoids have the power to reduce the expression of inflammatory mediators [94]. Also, ergosterol and peroxide-derived ergosterol from CV have been demonstrated to suppress the inflammatory response of lipopolysaccharide by lowering pro-inflammatory cytokines [95]. 

Our results revealed that GA3 significantly decreased the gene expression level of PPARα while increasing the activator protein 1 (AP-1) gene expression level. He et al. [96] stated that the expression of PPARα mRNA is markedly decreased in inflammatory liver disorders. Through transrepression of AP-1 and NF-κB signaling pathways, PPARα exerts anti-inflammatory actions. PPARα can successfully trans-repress a variety of pro-inflammatory gene promoters controlled by NF-κB or AP-1 response elements by protein-protein interactions [97]. The p65 and c-Jun components of the NF-κB and AP-1 transcription factors interact correspondingly with PPARα physically and functionally. Additionally, PPARα significantly lowers the gene production of pro-inflammatory cytokines in the liver, such as pro-IL-1 β, pro-IL-6, and pro-TNF [98]. By promoting the transcription of a number of pro-inflammatory genes, NF-κB and AP-1 play a crucial role in inflammation. The transcription factors NF-κB and AP-1 are stimulated as PPARα is reduced [99].

On the other hand, PPARα mRNA expression in the liver was remarkably more increased in the RJ supplemented group than in the GA3-intoxicated group, while the gene expression level of AP-1 was significantly decreased. Yoshida et al. [100] reported that RJ up-regulates the hepatic gene expression level of PPARα in diabetic mice. The inhibitory action of RJ on AP-1 gene expression is related to the up-regulation of PPARα expression since PPARα exerts anti-inflammatory effects through trans-repression of AP-1 [97]. Moreover, the up-regulation of the PPARα expression level observed in the RJ group explained the lower serum levels of NF-κB and TNF-α recorded in this group in agreement with previous literature proving that in models of systemic inflammation, non-alcoholic steatohepatitis, and atherosclerosis, PPARα may negatively affect the pro-inflammatory and acute phase response signaling pathway [101]. Also, PPARα activation boosts antioxidant defense and lowers oxidative stress [96].

Similarly, our findings reported the anti-inflammatory activity of CV supplementation via an elevated gene expression level of PPARα and lowered gene expression level of AP-1. Many transcription factors such as peroxisome proliferator-activated receptors and the retinoid X receptor (RXR) are stimulated by β-carotene, which is a bioactive component present in CV, as it is responsible for the production of retinol and retinoic acid [102].

Our recorded biochemical results are compatible with the histopathological alterations. Concerning the histological observations in the hepatic parenchyma of the GA3-treated group, there was severe cellular and nuclear degeneration, vacuolation, and apoptosis, besides dilatation and congestion of all hepatic vessels. in addition to lymphocytic infiltrations as previously revealed [46,62,103]. Vacuolar degeneration was recorded as one of the main first responses to cell injury [104]. Hepatocytic vacuolation is caused by oxidative changes and lipid peroxidation induced by GA3 [105]. Subsequently, lipid peroxides accumulated and produced organelles disintegration and membrane permeability alterations. Rahman and Mcnee [106] discussed that the inflammatory cell leakage was accompanied by cellular oxidation, in which the free radicals destruct the endothelial cells making output of interleukin and cytokine-induced neutrophil chemoattractant mediators, leading to filling of microcirculation with the inflammatory cells, which then go to the liver interstitium. Our results indicate that treatment with RJ has the power to recover the normal structure of hepatocytes and their secretions. The hepatoprotective effect of RJ was explained previously by Cemak et al. [107] and Mostafa et al. [28] as it preserves the integrity of hepatocyte membrane and prevents the hepatic enzymes leakage into the circulation. Sequentially, CV treatment makes partial hepatic improvement of the injured tissue. Kumar et al. [26] discussed the protective effect of CV, due to high carotenoid contents, which have anti-inflammatory and antioxidative activities. Also, Naguib [108] and El-Fayoumy et al. [109] found that the antioxidant properties of CV are due to its chemical constituents of active hydroxyl group plus unsaturated bonds that have a high ability to prevent cellular oxidation by recovering some free radicals.

In the control group, Crossmon’s trichrome-stained liver sections showed collagen fibers of a fine normal periportal distribution as revealed by Alshawsh et al. [110]. While, in the GA3-treated group, there was a massive periportal collagen fiber distribution. These results plus the significant area percentage of fibers signalize the initiation of fibrosis with the long administration of GA3. Bauer and Schuppan [111] explained that hepatic fibrosis is mainly stimulated by hepatocyte degeneration and necrosis, which causes Kupffer cells stimulation and production of cytokines and growth factors, which enhance the proliferation of stellate cells and excessive secretion of connective tissue fibers and matrix. Moreover, Ross and Pawlina [112] clarified that nuclear damage caused by lipid peroxidation enhances collagen formation. The administration of RJ and CV lead to minimizing fibrosis, which was proved by our result of area percentage for collagen fibers, which was significantly more decreased than in the GA3-treated group.

The glycogen and total protein contents in the PAS and Bromophenol blue-stained sections, respectively, were depleted significantly in response to GA3 administration compared to normal sections. Our results agree with those revealed by Ali et al. [103]. While the sections of GA3 + RJ and GA3 + CV-treated groups, which showed nearly normal contents, emphasize the hepatoprotective effect of RJ and CV.

COX-2 was expressed as a few granules in normal livers, mainly in the sinusoidal lining. Excessive COX-2 expression is accompanied by inflammation and tissue injury [113]. This is compatible with our result of COX-2 in the GA3-treated group as it was expressed in the inflamed hepatocytes, sinusoidal lining including endothelial and Kupffer cells mainly surrounding collagen proliferation and inflammatory cell infiltration. This agreed with that mentioned by Denda [114] who stated that the Kupffer cells were the main hepatic prostanoids producers. RJ reduces tissue damage through the reduction of TNF-α and COX-2 expression [113]. CV administration inhibits the COX-2 expression due to their inhibitory activity that blocks the inflammatory mediator’s formation by COX-2 inhibitors as discussed by Cheng et al. [115].

Mohammed et al. [116] previously stated that iNOS levels were increased in cirrhosis. Moreover, its expression increased mainly surrounding the areas of fibrosis [117]. The excessive iNOS expression in hepatic tissue treated with GA3 with increased fibrosis suggests that GA3 toxicity is associated with increased production of nitric oxide. RJ significantly decreased the iNOS expression through the reduction of inflammation [28,118]. CV treatment has an inhibitory action on iNOS production due to anti-inflammatory, antioxidant, and free radical scavenging effects, and the presence of chlorophyll [119].

## 5. Conclusions

Depending on our biochemical and histopathological results, GA3 induced liver damage as implied by the elevation of serum biochemical parameters, reduction of the antioxidant activity, and increase in the inflammatory mediators. Also, GA3 induced cytogenotoxicity as manifested by chromosomal abnormalities and abnormal mitotic index. However, treatment with RJ or CV was found to reduce GA3-induced cytogenotoxicity and hepatotoxicity. The hepato-protection was associated with the modulation of the PPARα/AP-1 signaling pathway, which plays a substantial role in diminishing oxidative stress and inflammation. Therefore, RJ and CV have promising therapeutic roles against cytogenotoxicity and liver toxicity manifested by GA3. So, we recommend using RJ or CV as food supplements for people living in areas where GA3 is used as a plant growth promotor to protect against GA3-induced toxicity.

## Figures and Tables

**Table 1:** Sequences of primers used in quantitative real-time polymerase chain reaction.

Gene	Sequence 5′–3′	Gene Accession Number
PPARα	Forward sequence: TTC GGA AAC TGC AGA CCT Reverse sequence: TTA GGA ACT CTC GGG TGA T	NC_051342.1
AP-1	Forward sequence: CAA CGC CTC GTT CCT CCC G Reverse sequence: GGC GCG GAG GTG CGG CTT C	NC_051341.1
β-actin	Forward sequence: AGG TCA TCA CTA TCG GCA AT Reverse sequence: ACT CAT CGT ACT CCT GCT TG	NC_051347.1

**Table 3:** Effect of RJ and CV on GA3-induced changes in liver function biomarkers in rats.

Groups	ALT (U/L)	AST (U/L)	ALP (U/L)	γGT (U/L)	Albumin (g/dL)	Total Bilirubin (mg/dL)
Control	75 ± 2	113 ± 13	219 ± 8	14 ± 1	4.3 ± 0.4	0.8 ± 0.1
RJ	69 ± 3	110 ± 6	214 ± 12	13 ± 1	4.7 ± 0.3	0.7 ± 0.1
CV	75 ± 5	113 ± 5	216 ± 12	13 ± 2	4.3 ± 0.4	0.75 ± 0.04
GA3	139 ± 11 ***	199 ± 7 ***	479 ± 16 ***	25 ± 2 ***	1.9 ± 0.1 ***	1.5 ± 0.1 ***
GA3 + RJ	85 ± 8 ^###^	129 ± 9 ^###^	378 ± 19 ***^###^	15 ± 1 ^###^	3.8 ± 0.2 ^###^	0.9 ± 0.1 ^###^
GA3 + CV	96 ± 3 *^###^	148 ± 2 **^###^	413 ± 5 ***^###^	17 ± 0.5 ^###^	3.3 ± 0.5 *^##^	1 ± 0.1 *^###^

Data are expressed as Mean ± SD (N = 6). ALT: alanine aminotransferase; AST: aspartate aminotransferase; ALP: alkaline phosphatase; γGT: gamma-glutamyl transferase. * *p* ≤ 0.05, ** *p* ≤ 0.01, *** *p* ≤ 0.001 vs. Control. ^##^ *p* ≤ 0.01, ^###^ *p* ≤ 0.001 vs. GA3.

**Table 4:** Effect of RJ and CV on GA3-induced changes in hepatic oxidant/antioxidant status in rats.

Groups	MDA (nmol/g)	SOD (U/g)	CAT (U/L)	GPx (U/g)
Control	1.3 ± 0.2	2.1 ± 0.5	2.9 ± 0.2	2 ± 0.1
RJ	1.1 ± 0.1	2.2 ± 0.2	2.9 ± 0.2	2.3 ± 0.2
CV	1.3 ± 0.3	2.2 ± 0.1	2.9 ± 0.2	2 ± 0.1
GA3	2.6 ± 0.2 ***	0.72 ± 0.03 ***	0.8 ± 0.02 ***	0.8 ± 0.1 ***
GA3 + RJ	1.6 ± 0.1 ^###^	1.82 ± 0.04 ^###^	2 ± 0.1 ***^###^	1.7 ± 0.2 ^###^
GA3 + CV	1.8 ± 0.1 *^###^	1.77 ± 0.04 ^###^	2 ± 0.2 ***^###^	1.6 ± 0.2 *^###^

Data are expressed as Mean ± SD (N = 6). MDA: malondialdehyde; SOD: superoxide dismutase; CAT: catalase; GPx: glutathione peroxidase. * *p* ≤ 0.05, *** *p* ≤ 0.001 vs. Control. ^###^ *p* ≤ 0.001 vs. GA3.

## Data Availability

All data analyzed during this work are included in the published article. Raw data are available on request from the corresponding author.
